# CRISPR/Cas9-mediated multiple gene editing in *Brassica oleracea* var. *capitata* using the endogenous tRNA-processing system

**DOI:** 10.1038/s41438-018-0107-1

**Published:** 2019-02-01

**Authors:** Cunfa Ma, Chenzeng Zhu, Min Zheng, Mengci Liu, Dejun Zhang, Baoli Liu, Qinfei Li, Jun Si, Xuesong Ren, Hongyuan Song

**Affiliations:** Key Laboratory of Horticulture Science for the Southern Mountains Regions, Ministry of Education; College of Horticulture and Landscape Architecture, Southwest University, 400715 Chongqing, China

**Keywords:** Molecular engineering in plants, Gene targeting

## Abstract

Cabbage (*Brassica oleracea* var. *capitata*) is a biennial plant with strong self-incompatibility and an obligate requirement for prolonged vernalization by exposure to low temperatures to induce flowering. These characteristics significantly increase the difficulty of exploiting novel germplasm induced by physical or chemical mutagens. In this study, we report a CRISPR/Cas9 gene-editing system based on endogenous tRNA processing to induce high efficiency and inheritable mutagenesis in cabbage. Using the phytoene desaturase gene *BoPDS*, the S-receptor kinase gene *BoSRK*, and the male-sterility-associated gene *BoMS1* as the target genes, multisite and multiple gene mutations were achieved using a construct with tandemly arrayed tRNA-sgRNA architecture to express multiple sgRNAs. The *BoSRK3* gene mutation suppressed self-incompatibility completely, converting the self-incompatible line into a self-compatible line. In addition, the *BoMS1* gene mutation produced a completely male-sterile mutant, which was highly cross compatible with its nonmutant isoline at the flowering stage as a result of a simultaneous *BoSRK3* gene mutation, enabling the economic propagation of the male-sterile line through bee-mediated cross-pollination. Interestingly, higher site mutation efficiency was detected when a guide sequence was inserted into a location in the tandemly arrayed tRNA-sgRNA architecture that was distal from the upstream Pol III promoter. In addition, mutation sites were also detected in the paralogous genes of the *BoPDS* and *BoSRK* genes that had fully consistent sequences or base mismatches but beyond the “seed” region in the spacer sequence compared with the target sgRNAs. Collectively, our results demonstrate that the CRISPR/Cas9 system, coupled with an endogenous tRNA-processing system, is an efficient tool to improve cabbage traits.

## Crop genetics: CRISPR editing of multiple genes in cabbage

Researchers in China have shown that the gene-editing system CRISPR/Cas9 can be used to efficiently mutate genes of interest in cabbage. Cabbage plants normally do not self-pollinate, making the use of traditional mutagens difficult. Hongyuan Song of Southwest University therefore turned to CRISPR/Cas9, a system which can introduce precise mutations into specific genes. Song’s team targeted three genes: one related to coloration, another to self-incompatibility, and a third involved in pollen development. They began by mutating each gene separately to test the system’s efficiency. Next, they built a CRISPR/Cas9 construct that would simultaneously mutate the pollen gene and the self-incompatibility gene. One-third of the plants produced using the construct had mutations in both of the target genes. These findings demonstrate that CRISPR/Cas9 is a valuable tool for trait improvement and genetic research in cabbage.

## Introduction

Methods for rapidly and efficiently editing plant genomes are useful for research into gene function and crop improvement. In the past decades, chemical or physical mutagenesis methods were extensively used to produce random mutations that were subsequently screened for the phenotypes of interest^1^. Compared with such traditional random mutagenesis, which is inefficient and laborious, targeted gene editing technologies can markedly improve the process of creating mutants. In recent years, methods involving sequence-specific nucleases to create targeted DNA double-stranded break (DSB) genetic tools, such as zinc-finger nucleases and transcription activator-like effector nucleases (TALENs), were used for genome editing^2–4^. Given the substantial technical complexity and the experience required to design and assemble the gene constructs, these methods were not widely used^1,3^. Recently, a new gene-editing tool, the type II clustered regularly interspaced short palindromic repeats (CRISPR)-associated protein (Cas) system (CRISPR/Cas9) with its high efficiency, low cost, simple design, and versatility has been demonstrated to be efficient for gene disruption in many plant species, including *Arabidopsis*, *Nicotiana benthamiana*, *N. tabacum*, rice, wheat, maize, sorghum, tomato, potato, sweet orange, poplar, and liverwort^1–3,5^.

Many important traits are often regulated by multiple genes or paralogous genes. To improve such traits requires the editing of multiple genes. Fortunately, the Cas9 protein can be directed to specific sites by different single-guide RNAs (sgRNAs) that provide an opportunity to achieve multiplex gene editing by expressing Cas9 along with the multiple sgRNAs^6,7^. In plants, the most common strategy is to stack multiple independent sgRNA-expressing cassettes in a single construct and to deliver the construct into plant cells^6,8^. However, this sgRNA-expressing strategy is challenging for most organisms due to the limitations of the delivery method and the vector capacity. By using a synthetic gene with tandemly arrayed tRNA-sgRNA architecture, multiple sgRNAs were efficiently and precisely produced in vivo in rice by the endogenous RNases, RNase P and Z, for the first time^9^. In addition, the tRNA-processing sgRNA expression system was successfully demonstrated in other organisms^10–13^.

The CRISPR/Cas9 system is becoming a powerful tool for genome editing in plants^2,5^, but, in *Brassica*, only a few successful instances of genome editing have been reported^7,14–16^. One of these cases was the *GA4* gene knockout in a doubled-haploid genotype AG DH1012 (a broccoli-like Brassica) from the *Brassica oleracea* var. *alboglabra* (A12DHd) × *B*. *oleracea* var. *italica* (Green Duke GDDH33) mapping population^7^. Cabbage (*Brassica oleracea* var. *capitata*) is an important cruciferous leafy vegetable crop that is planted worldwide. With the availability of the cabbage genome sequence^17^, the CRISPR/Cas9 system may enable the exploration of gene functions and/or the improvement of the traits of cabbage varieties using specific gene modification.

Self-incompatible lines with different S-alleles are usually employed to breed cabbage F_1_ hybrids. However, unstable self-incompatibility problems^18^ prompt increasing numbers of breeders to breed cabbage hybrids using the male-sterility approach^19^. Seed purity can be guaranteed when producing F_1_ hybrid seeds using the male-sterile line, but hybrid seed production cost is increased using this route, because both the maintainer line and the male-sterile line have to be propagated manually at the bud stage due to the strong self-incompatibility. The S-receptor kinase (*SRK*) gene is the key stigma determinant of cabbage self-incompatibility^20^. Disrupting self-incompatibility by mutating the *SRK* gene to generate a self-compatible inbred line should be feasible to reduce the seed production cost of the cabbage F_1_ hybrids using the male-sterility approach.

Plant male reproductive development involves stamen meristem specification to achieve pollen grain formation and pollination events; defects in any of these events can lead to male sterility, permitting the generation of valuable male-sterile lines for hybrid breeding^21^. A plant homeodomain-finger family of MALE STERILITY1 (MS1) transcription factors was shown to be involved in the regulation of male sterility by affecting tapetal and pollen wall development in *Arabidopsis*^22^. In addition, the *PERSISTANT TAPETAL CELL1*(*PTC1)* gene in rice, the *HvMALE STERILITY1* (*HvMS1*) gene in barley, and the *CA05g06780* gene in pepper, all with similar amino acid sequences to the *MS1* gene in *Arabidopsis*, had been found to be involved in the regulation of male gametogenesis^23–25^. The *Bol035718* gene (from the *Brassica* database) in cabbage has an amino acid sequence highly similar to that of the *MS1* gene in *Arabidopsis* and is involved in pollen development as verified by RNAi technology in cabbage and genetic complementation analysis in *Arabidopsis thaliana* (data not shown). We propose that a male-sterile cabbage mutant could be generated using the CRISPR/Cas9 system to modify the *BoMS1* gene.

In this study, we synthesized a tandemly arrayed tRNA-sgRNA sequence to simultaneously produce numerous sgRNAs using plant endogenous tRNA processing. The phytoene desaturase gene *BoPDS*, the self-incompatibility determinant gene *BoSRK3*, and the *BoMS1* gene associated with male sterility were selected as the target genes to investigate genome editing in cabbage separately or simultaneously. We showed that the CRISPR/Cas9 system successfully knocked out the target genes using the tRNA-processing system-based methods. In addition, the cabbage male-sterile line caused by the *BoMS1* gene mutation could be economically propagated using the *BoSRK* gene mutant. Our results demonstrate that the CRISPR/Cas9 system with endogenous tRNA processing is a powerful tool to improve cabbage varieties using specific gene modification.

## Results

### Efficient mutation of the *BoPDS* gene via the tRNA-processing system

**Fig. 1:**
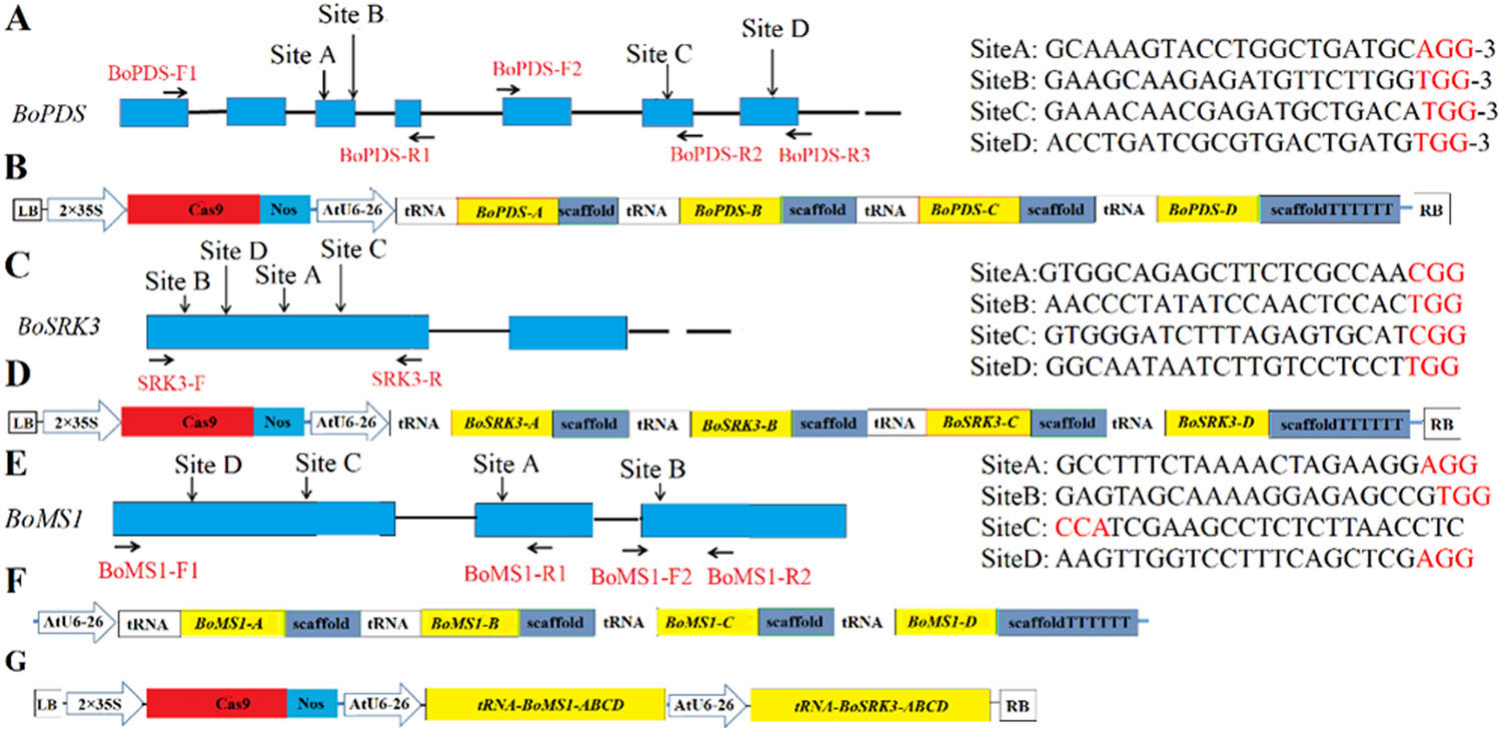
Vector construct for genome editing of the *BoPDS, BoSRK3* and *BoMS1* genes. **a**, **c**, **e** Diagram of the cabbage *BoPDS*, *BoSRK3*, and *BoMS1* genes, respectively, with the four target sites indicated. **b**, **d** Diagram illustrating the engineered CRISPR/Cas9 vector with the tRNA-processing system based on multiplex sgRNAs for the *BoPDS* and *BoSRK3* genes. **f** Diagram illustrating the tRNA-sgRNA intermediate vector for the *BoMS1* gene. **g** Diagram illustrating the engineered CRISPR/Cas9 vector for the *BoMS1* and *BoSRK3* gene editing. The blue boxes indicate exons, and the black lines indicate introns. The target sequence is shown in black letters. PAM is marked in red letters. The primer sites used to evaluate the mutation types are shown by black arrows. The screening marker gene expression cassette of *Bar* was not displayed in the T-DNA region (**b**, **d**, **g**)

PDS (phytoene desaturase) is a key enzyme in the carotenoid biosynthesis pathway, and plants with a mutated *PDS* gene should show an albino phenotype that can be easily recognized^26^. The cabbage phytoene desaturase gene *BoPDS* was selected as the target gene to first explore the genome modification efficiency using the tRNA-processing system. Four target sites with the protospacer adjacent motif (PAM) (NGG) on their 3′-ends in the *BoPDS* gene were manually selected. The A and B sites were both located in the third exon, while the C and D sites were located in the sixth and seventh exons, respectively (Fig. 1a). The A, B, C, and D target site sequences were inserted into the *Bbs*I, *Bsa*I, *Bsm*BI, and *Bfu*AI sites of the synthetic tRNA-sgRNA vector, respectively. The sgRNA-expressing cassette was cloned into the pCACas9 vector as a *Bam*HI and *Eco*RI fragment to generate the pCACas-tRNA-sgRNA-PDS-ABCD vector (Fig. 1b).

**Fig. 2:**
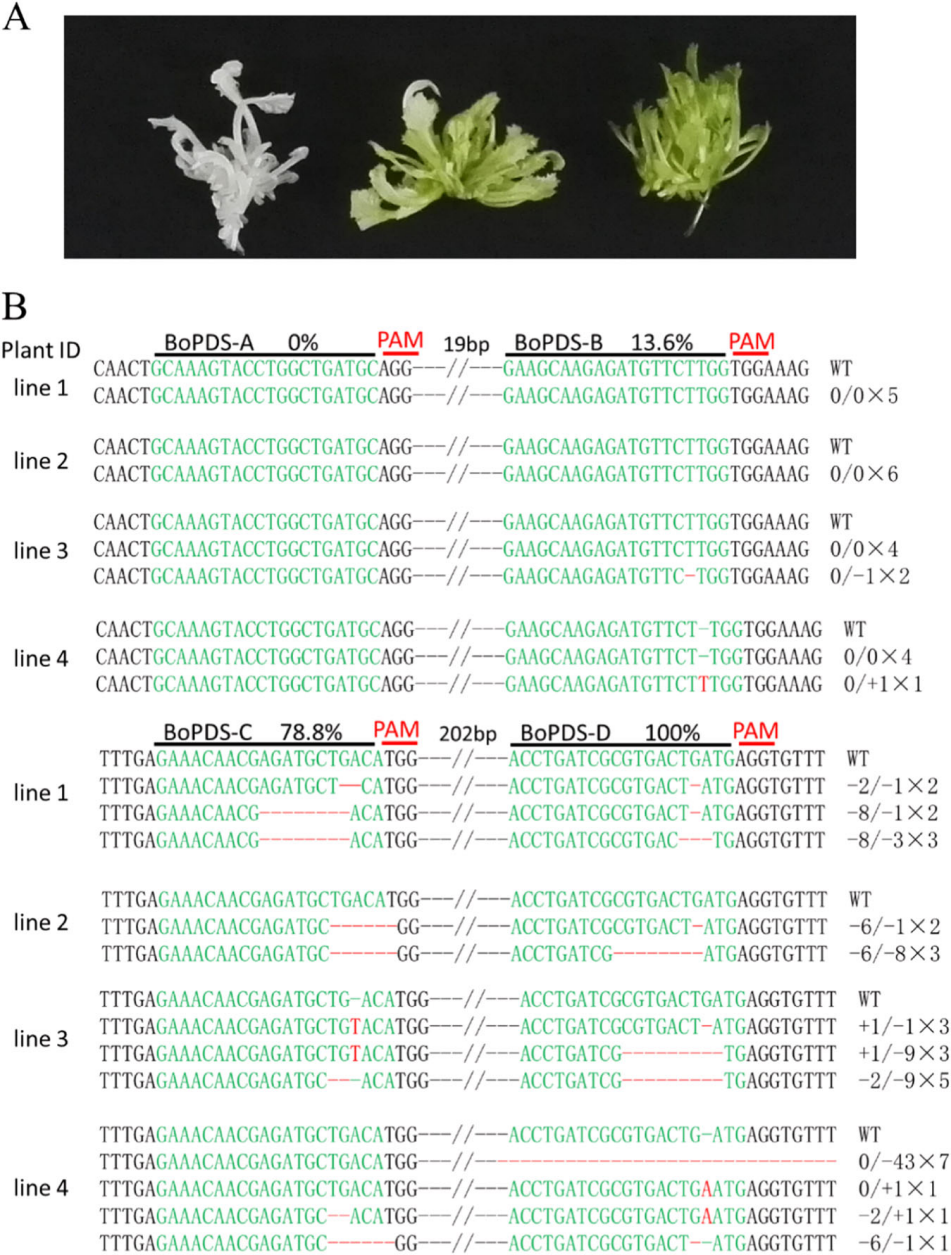
Site mutagenesis in the *BoPDS* gene using the tRNA-processing system. **a** Albino phenotype of the *PDS* mutants. Left, albino shoots. Center, mosaic shoots. Right, wild-type shoots. **b** Targeted mutagenesis of *BoPDS*. Sequence alignments of the A, B, C, and D target sites in four *BoPDS*-mutated plants. The target sequences are shown in green with mutations in red

Twenty-five T0 lines were generated for the pCACas-tRNA-sgRNA-PDS-ABCD vector transformation. Among them, ten lines exhibited the completely albino phenotype, while seven lines were chimeras, exhibiting a mosaic albino phenotype, resulting in 68% knockout efficiency based on the abnormal phenotypes (Fig. 2a). PCR was performed on three completely albino plants and one chimeric albino plant using the primers BoPDS-F1 and BoPDS-R1 flanking the A and B target sites and BoPDS-F2 and BoPDS-R3 flanking the C and D target sites of the *BoPDS* gene (Fig. 1a). The PCR products were cloned and sequenced to investigate the mutation types and frequencies for the individual sites. For target site A, no mutagenesis was detected in any of the lines. Heterozygous mutations with a 1 bp deletion or insertion were only detected in lines 3 and 4 for target site B. For target site C, biallelic mutations in lines 1 and 3, mono-allelic homozygous mutations with a 6 bp deletion in line 2, and a chimeric mutation with a 2 or 6 bp deletion in line 4 were detected. For the target site D, biallelic mutations in lines 1, 2, and 3, and a chimeric mutation in line 4 were detected (Fig. 2b). Although four sites in the *BoPDS* gene were simultaneously targeted, no large DNA fragment deletions were detected by further PCR analysis in any of the 17 transgenic plants (Supplementary Fig. 1A). In addition, the mutation efficiencies at the A, B, C, and D sites increased in order from A to D, a phenomenon that was positively correlated with the distance from the upstream *U6* promoter in the guiding sequence position of the tandemly arrayed tRNA-sgRNA architecture (Figs. 1b and 2b).

### The *BoSRK* gene mutation suppressed self-incompatibility completely

Based on the *BoSRK3* gene sequence of the “F416” inbred line (*SRK3* S-haplotype), four target sites sequence located in the first exon were inserted into the *Bbs*I, *Bsa*I, *Bsm*BI, and *Bfu*AI sites of the tRNA-sgRNA vector (Fig. 1c). A pCACas-tRNA-sgRNA-SRK-ABCD vector was generated by inserting the sgRNA-expressing cassette into the pCACas9 vector (Fig. 1d).

**Fig. 3:**
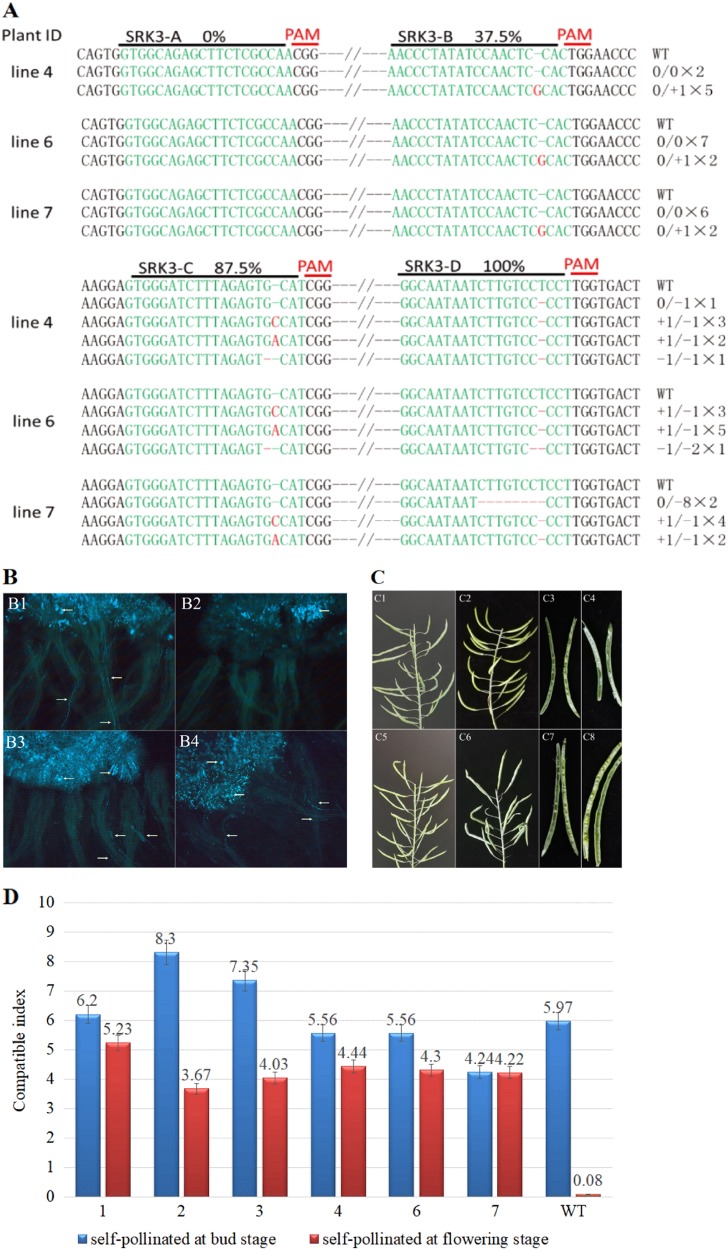
Targeted mutagenesis of the *BoSRK3* gene (X79432.1) by tRNA-processing system-based editing. **a** Summary of the mutation types and frequencies in sites A–D. The target sequences are shown in green, and the mutations are shown in red. **b** In situ fluorescence microscopy of pollen germination on the stigma of the mutant plants and wild-type plants. b1 Bud stage self-pollination of the wild-type plants. b2 Flowering stage self-pollination of the wild-type plants. b3 Bud stage self-pollination of the mutant plants. b4 Flowering stage self-pollination of the mutant plants. White arrows indicate pollen grain germination on the stigma and pollen tube growth down the style. **c** Silique growth and seed setting in the mutant and wild-type plants. c1 and c3 Silique growth and seed setting of the wild-type plants after the bud stage self-pollination. c2, c4 Silique growth and seed setting of the wild-type plants after flowering stage self-pollination. c5, c7 Silique growth and seed setting of mutant plants after the bud stage self-pollination. c6, c8 Silique growth and seed setting of mutant plants after the flowering stage self-pollination. **d** Investigation of the self-compatibility index of the mutant and wild-type plants at the bud or flowering stages; self-compatibility index = number of seeds/number of flowers. Bars represent the mean ± standard deviation (SD)

A total of 29 Cas9-positive T0 generation pCACas-tRNA-sgRNA-SRK/ABCD lines were obtained using *Agrobacterium*-mediated transformation. As with the results of the *BoPDS* gene editing, no large DNA fragment deletions occurred among the 29 lines (Supplementary Fig. 1C), but all the lines showed successful gene editing estimated by the direct target region sequencing chromatogram. As with the results from the *BoPDS* gene, we did not detect any mutations at site A of the *BoSRK3* gene in any of the lines using clone sequencing, but a heterozygous mutation with a G nucleotide insertion was detected at site B in all the lines. Only chimeric mutations were detected at site C in all the lines, but mono-allelic homozygous mutations with a 1 bp deletion in line 4 and biallelic mutations in lines 6 and 7 were discovered at site D (Fig. 3a). In addition, the mutation efficiency at the A, B, C, and D sites in the *BoSRK3* gene was positively associated with the distance between the position of the specific guiding sequence located in the tandemly arrayed tRNA-gRNA architecture and the upstream *U6-26* promoter (Figs. 1d and  3a), the result was similar to that from the *BoPDS* gene modification.

**Fig. 4:**
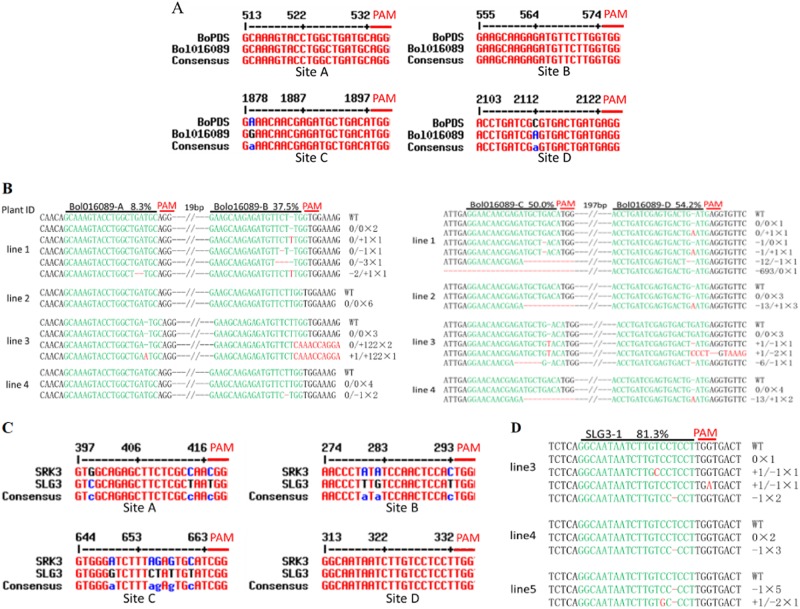
Paralogous gene mutations mediated by the CRISPR/Cas9 system. **a** Site A, B, C, and D sequence alignment between the *Bol016089* and *BoPDS* genes. **b** Summary of the mutation types and frequencies at sites A–D in the *Bol016089* gene. **c** Sites A, B, C, and D sequence alignment between the *BoSLG3* gene (X79431.1) and the *BoSRK3* gene. **d** Summary of the mutation types and frequencies at site D in the *BoSLG3* gene. The number of each mutation type was used to estimate the genome editing efficiency. The target sequences are shown in green and the mutations in red

The degree of self-compatibility of the *BoSRK3* gene mutants was estimated by the observation of pollen grain germination on the stigma surface using fluorescence microscopy and a self-compatibility index investigation. A large number of pollen grains germinated on the stigma and formed pollen tubes penetrating through the style of “F416” after self-pollination at the bud stage (Fig. 4b1), but no evident elongated pollen tube growing through the style was observed after self-pollination at the flowering stage (Fig. 4b2). Extended seed siliques and developed seeds of “F416” were observed after self-pollination at the bud stage (Fig. 4c1, c3) but not at the flowering stage (Fig. 4c2,  c4) demonstrating that “F416”is a strongly self-incompatible line. As expected, there were obvious signs of pollen grain germination on the stigma surface and pollen tube growth through the style in the *BoSRK3* gene mutant at both the bud and flowering stages (Fig. 4b3–b4), while extended seed siliques with full seeds were observed in the *BoSRK3* gene mutant after self-pollination at the bud and flowering stages (Fig. 4c5–c8). The self-compatibility index of the mutants was similar to or slightly lower than that of “F416” self-pollinated at the bud stage, but the index exhibited by all mutant plants at the flowering stage was significantly higher than that of “F416” self-pollinated at the same stage (Fig. 4d). The results demonstrated that the *BoSRK* gene mutation in “F416” completely suppressed the self-incompatible phenotype.

### Mutations were detected in paralogous genes of the target genes

It has been shown that the CRISPR/Cassystem cleaves genomic DNA sequences containing mismatches to the single-guide (sgRNA) strand. Another gene, *Bol016089*, in the cabbage genome (from *Brassica* genome database) had a sequence highly similar to that of the *BoPDS* gene; the nucleotide sequences of the A and B sites in the *Bol016089* gene were completely paired with the A and B sites in the *BoPDS* gene, but there was a base mismatch for both the C and D sites in the *Bol016089* gene (Fig. 4a). Although no mutation was detected in the *BoPDS* gene at site A in any of the lines (Fig. 2b), we detected one cloned mutation at site A of the *Bol016089* gene in lines 1 and 3, resulting in 8.3% editing efficiency (Fig. 4b). In addition, an editing efficiency of 37.5% was found at site B and is higher than the editing efficiency at site B in the *BoPDS* gene (13.6%) (Figs. 3b and 4b). Because of one base mismatch with the leader sequence in the sgRNA, lower editing efficiencies were detected at sites C (50%) and D (54.2%) in the *Bol016089* gene compared with the equivalent editing efficiencies at sites C (78.8%) and D (100%) in the *BoPDS* gene (Figs. 4b and  3b). Although a 693 bp fragment was deleted at the site C region in line 1, and a 122 bp fragment was inserted into the site B region in line 3 (Fig. 4b), no obvious fragment deletions in line 1 or fragment insertions in line 3 were detected using PCR (Supplementary Fig. 1B, lanes 1 and 3).

The nucleotide sequence of the receptor domain on the *SRK* gene showed a high sequence similarity with that of the S-locus glycoprotein (*SLG*) gene from the same haplotype, which is another gene involved in the regulation of self-incompatibility, and both had been proven to be a single copy gene^20^. Four sites with nucleotide sequences highly similar to those of the *BoSLG3* gene from “F416” were found in the A, B, C, and D target sites of the *BoSRK3* gene. There were 2–6 bp mismatches in the A, B, and C sites in the *BoSLG3* gene compared with the site sequence in the *BoSRK3* gene; only the D site sequence was identical to that of the D target site in the *BoSRK3* gene (Fig. 4c). We did not detect any mutation at the A, B, or C target sites of the *BoSLG3* gene in any of the transgenic plants. However, mutations were detected in 10 lines of the D site of the *BoSLG3* gene using PCR and clone sequencing (Fig. 4d).

### Simultaneous mutation of the *BoMS1* and *BoSRK3* genes to produce a cabbage male-sterile line that can be propagated economically for F_1_ variety seed production

The simultaneous mutation of multiple genes in the same cell or individual has many potential applications, such as an important trait that is regulated by multiple functionally related genes. To reduce the cost of seed production of the cabbage male-sterile line for F_1_ hybrid seed propagation, it is necessary to mutate the self-incompatibility regulation gene in the cabbage male-sterile line. We assembled two tRNA-sgRNAs that targeted the *BoMS1* and *BoSRK* genes into the same vector to create a male-sterile cabbage mutant and to explore the economic feasibility of producing inbred seed of the cabbage male-sterile line.

**Fig. 5:**
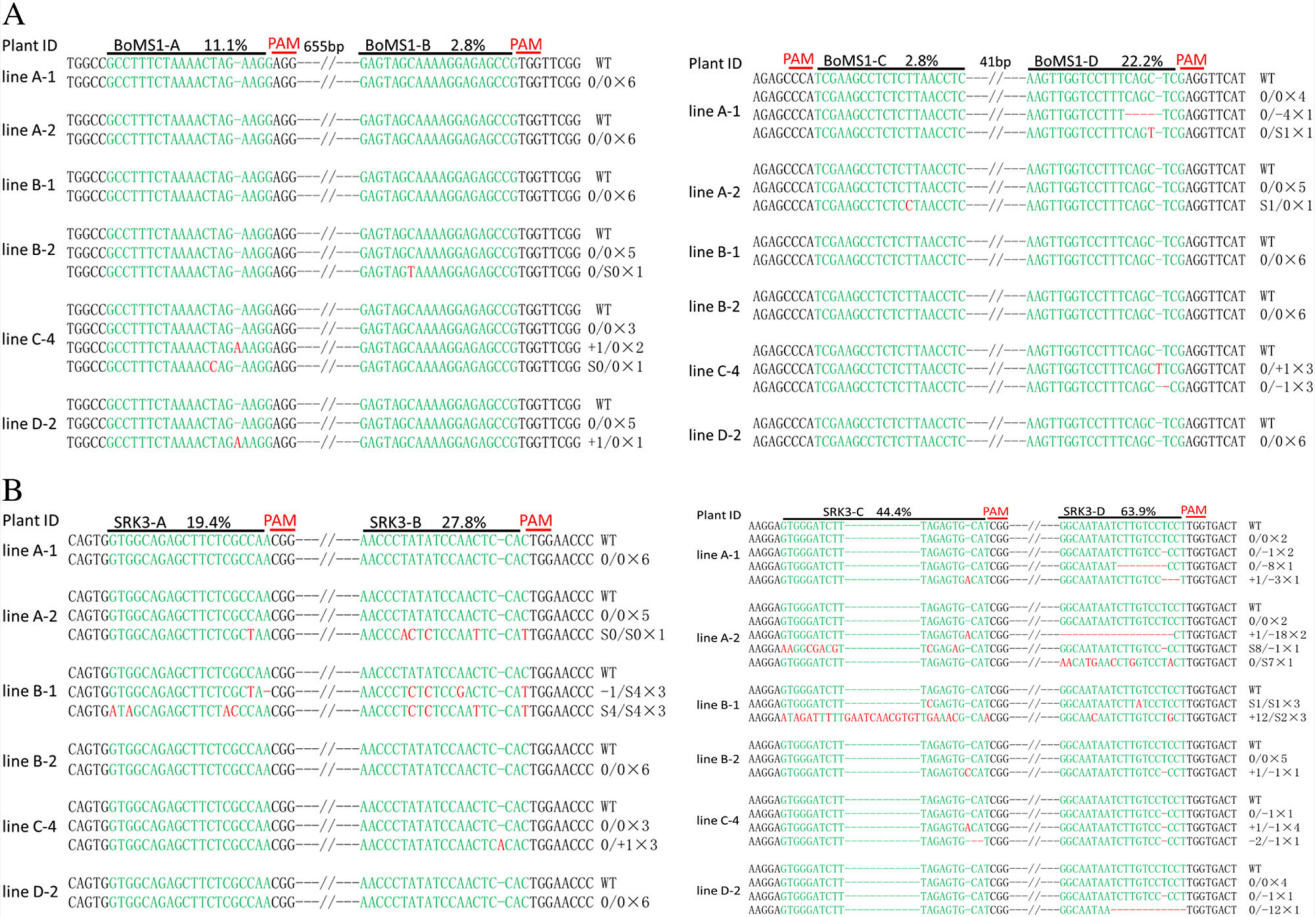
Mutation types and frequencies at sites in the *BoMS1* and *BoSRK3* genes in the double mutants. **a** Summary of the mutation types and frequencies at sites A–D in the *BoMS1* gene. **b** Summary of the mutation types and frequencies of sites A–D in the *BoSRK3* gene. The target sequences are shown in green and the mutations in red

A total of 18 *Cas9* gene-positive T0 lines for the PCACas-tRNA-sgRNA-SRK-ABCD/MS1-ABCD vector were identified. The mutation rate was calculated by direct sequencing of the target region of the *BoMS1* and *BoSRK* genes for each line. The *BoMS1* gene in six transgenic plants had an overlap peak at the target sites. However, mutations in the *BoSRK3* gene were found in 13 transgenic plants. The mutation efficiency of the *BoSRK3* gene was clearly higher than that for the *BoMS1* gene mutation but markedly lower than the mutation efficiency induced when only the *BoSRK3* gene was targeted (100%). The frequency and type of mutation at each target site in the *BoMS1* gene in the different plants were analyzed by clonal sequencing. Unexpectedly, we failed to detect any clonal mutations at any of the four target sites of *BoMS1* gene in the B-1 line in which the *BoMS1* gene mutation had been detected by direct target region sequencing. We speculated that the overlapping peak of sequencing chromatograms at the target sites of the *BoMS1* gene in the B-1 line should result from the purity of the PCR products and the quality of the sequence reads, because no mutation was detected by further Sanger sequencing of the *BoMS1* gene in 20 T1 plants derived from the B-1 line (data not shown). Fortunately, the *BoSRK3* gene mutations were detected in five other plants that harbored the *BoMS1* gene mutation, so the simultaneous mutation efficiency of both genes was 27.78% (Table 1). Nucleotide deletions, insertions, and substitutions were detected at different target sites of the *BoMS1* gene in the other five lines. The mutation frequency at the A site over all the plants was 11.11% (Table 1; Fig. 5a), while the lowest mutation frequency (2.78%) was detected at the B and C sites, because only one clone out of 36 was found with a single base substitution. However, a 22.22% mutation frequency was detected at the D site and was significantly higher than those at the A, B, and C sites (Table 1; Fig. 5a). In lines A-1, A-2, B-2, and D-2, only one mutation was detected as a heterozygous or chimeric mutation. However, mutations were detected at both the A and D sites in the C-4 line, and the D site had a biallelic mutation (Fig. 5a). For the *BoSRK3* gene, the mutation frequency was 19.44% with the mutation at the A site in lines A-2 and B-1 occurring as heterozygous and biallelic mutations, respectively. A mutation frequency of 27.78% was detected at the B site in all six lines in the form of biallelic mutations (line B-1), heterozygous mutations (lines A-2 and C-4), and wild type (lines A-1, B-2, and D-2). A higher mutation frequency (44.44%) was detected at the C site than at the A and B sites in the form of biallelic mutations (line B-1 line), heterozygous mutations (lines A-1 and B-2), chimeric mutations (A-2 and C-4 lines) and wild type (line D-2). A mutation frequency of 63.89% at the D site was detected in the form of a chimeric mutation (lines A-1, A-2 and D-2), a heterozygous mutation (line B-2), a biallelic mutation (line B-1) and mono-allelic homozygous mutations (line C-4) (Table 1; Fig. 5b). In addition, more than one nucleotide substitution was detected at different sites of the A-2 and B-1 lines, which were clearly different from the single nucleotide substitution in the A-1, B-2, and C-4 lines (Fig. 5b). Among the four target sites of the *BoSRK3* gene, the mutation frequency increased in order from site A to site D (Table 1; Fig. 5b), with the mutation frequencies at the different sites similar to those from the individual *BoSRK3* gene modifications (Fig. 3a).

**Table 1:** Mutation efficiency in the T0 generation lines and in different sites of the *BoMS1* and *BoSRK3* genes

Target genes	No. of plants examined	No. of plants with mutation	Mutation efficiency (%)	No. of plants with double gene mutation	Double gene mutation efficiency (%)	Site A mutation frequency (%)	Site B mutation frequency (%)	Site C mutation frequency (%)	Site D mutation frequency (%)
*BoMS1*	18	5	27.78	5	27.78	11.11	2.78	2.78	22.22
*BoSRK3*	18	13	72.22	5	27.78	19.44	27.78	44.44	63.89

**Fig. 6:**
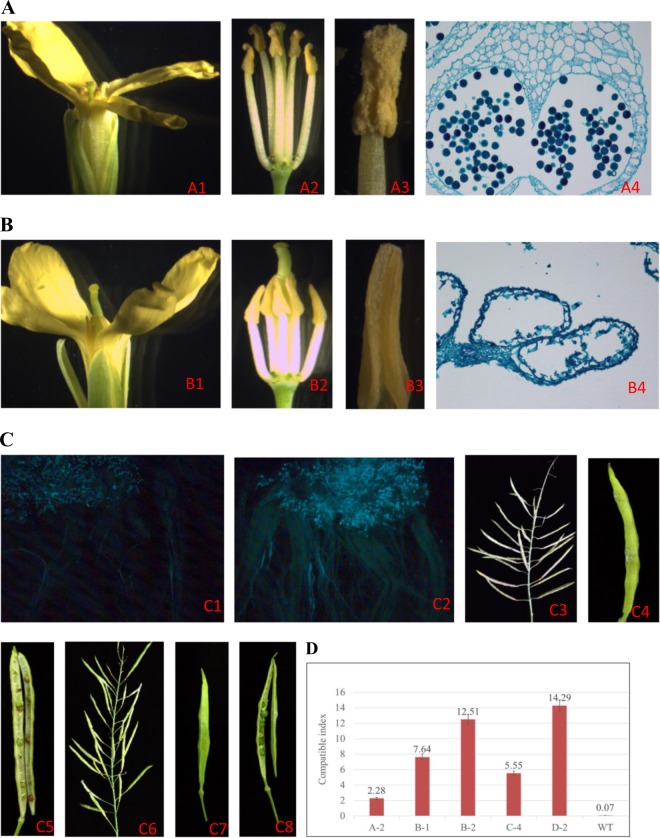
Floral organ morphology, development of the pollen and cross-compatibility of the wild type and the C-4 mutant. **a**, **b** Comparison of the lower, stamen, pistil, anther, and pollen development between the wild type and the C-4 mutant. a1–a3 floral organ morphology of the wild type; b1–b3 floral organ morphology of the C-4 mutant; a4 pollen formation in the anther of the wild type (10 × 20); b4 empty pollen sac in the anther from the C-4 mutant (10 × 20). **c** cross-compatibility between the C-4 mutant and the wild type with the same S-allele. c1 in situ fluorescence microscopy of pollen (from the wild type) germination on the stigma of the C-4 mutant at the bud stage; c2 in situ fluorescence microscopy of pollen (from the wild type) germination on the stigma of the C-4 mutant at the flowering stage; c3–c5 silique growth and seed-set of the C-4 mutant × wild type at the bud stage; c6-c8 silique growth and seed set of the C-4 mutant plant × wild type at the flowering stage. **d** Self- or cross-compatibility indices of the *BoMS1* and *BoSRK3* gene mutant plants. Bars represent the mean ± standard deviation (SD)

T0 plants with simultaneous mutations in both the *BoMS1* and *BoSRK* genes were transferred to soil and grown to anthesis (the A-1 line died after being planted). Plants of the other five lines proved to not be phenotypically different from the wild-type plants at the vegetative stage. After flowering, we found that only the C-4 line exhibited complete male sterility; the filaments becoming shorter, and the anthers failed to produce viable pollen, but the style of line C-4 was indistinguishable from that of the wild type (Fig. 6a, b: a1–a3 show the wild type; b1–b3 show the C-4 mutant). The phenotype of pollen development was examined using light microscopy. Unlike the situation in the wild type, the microspores and the tapetum of the C-4 mutant degenerated to form a mass of undifferentiated cells; ultimately, the anther locule was completely empty with no pollen development (Figs. 6a4 and b4). The cross-compatibility at the flowering stage between the C-4 plant and the wild type plant was determined by examining different traits, including pollen germination and the cross-compatibility index. A mono-allelic homozygous mutation of the *BoSRK3* gene in the C-4 plant was detected (Fig. 5b). As expected, a large number of pollen grains that germinated on the stigma and bundles of the pollen tubes were observed in the style of the C-4 plant pollinated by pollen grains from the wild type plant at the flowering stage, a result that was not obviously different from the situation with pollination at the bud stage (Fig. 6c1, c2). We found that the C-4 plant had a markedly elongated silique with a normal amount of seeds following pollination by wild type pollen at the flowering stage or at the bud stage (Fig. 6c3–c8). The cross-compatibility index between the C-4 male-sterile plant and the wild type maintainer line reached 5.56, indicating that the *BoMS1* gene mutation only affected the male gamete development with no negative effect on the female gamete (Fig. 6d). The results demonstrated that simultaneous *BoSRK3* gene mutations could release the cross-incompatibility obstacle between the cabbage male sterile and maintainer lines at the flowering stage. The self-compatibility index at the flowering stage of the other four fertile lines with the *BoSRK3* gene mutation, specifically lines A-2 (2.28), B-1 (7.64), B-2 (12.51), and D-2 (14.29), was much higher than for the wild type plant (0.07), although considerable variability was observed among the four plants (Fig. 6d). Even without the intercession of bees or artificial pollination in a space shielded with a fly net, some male-fertile plants showed very high seed-setting ability (Supplementary Fig. 2).

### Transmission of Cas9-induced mutations from the T0 to the T1 and F1 generations

To determine whether the Cas9-induced mutations were transmitted to the next generation, 20 T1 plants derived from a T0 mutant plant (line B-1) with only the *BoSRK3* gene mutation and 20 F1 plants derived from the cross between C-4 line (*BoMS1* and *BoSRK3* gene mutation) and the wild type were genotyped using PCR and amplicon Sanger sequencing. Line B-1 was a biallelic mutant at four target sites by deletion, substitution, and insertion in the *BoSRK3* gene (Fig. 5b). The individual plant sequencing results confirmed that these mutations were inherited by plants of the T1 generation in homozygotes and heterozygotes (Supplementary Fig. 3-A, A1–A2). In addition, a few new mutations were also detected in the T1 generation of the B-1 line. According to the overlapping sequencing chromatograms, new mutations at the site A were detected in 11 plants that were different from the mutations observed in the parental genome (Supplementary Fig. 3-A, A3). The similar phenomena were also found in *B. napus*^16^.

The T0 C-4 line could not be self-pollinated to generate T1 generation plants. Thus, it was crossed with “F416” to produce the F1 generation. The T0 C-4 line had biallelic mutations at site D due to a “T” nucleotide deletion and a “T” nucleotide insertion (Fig. 5a). The sequencing results confirmed that the two types of mutations were inherited. With the exception of one plant, the sequencing chromatograms in the 19 F1 plants had chaotic peaks at the target site (Supplementary Fig. 3-B, B1–B2), indicating that there were hemizygous mutations in the *BoMS1* gene at the target site in the 19 plants. There were 16 plants that had a “T” nucleotide deletion; 3 plants had a “T” nucleotide insertion at site D of the *BoMS1* gene, and no mutation was detected at site D in one plant (Supplementary Table 2). In addition, a heterogeneous “A” nucleotide insertion was simultaneously detected at site B of the *BoSRK3* gene in the T0 C-4 line (Fig. 5b), and we found that 11 plants had an “A” nucleotide insertion at site B in the *BoSRK3* gene after sequencing the amplicons (Supplementary Fig. 3-B, B4; Table 2). In total, 11 F1 plants of C-4 × WT inherited both the mutations of the *BoMS1* gene (site D) and the *BoSRK3* gene (site B).

The presence of the transgene (T-DNA) region was also examined using Cas9 gene PCR in the T1 and F1 populations. The absence of the T-DNA region was determined to be parallel in the PCRs negative for the *Cas9*, *sgRNA*, and *Bar* genes. The results indicated that four transgene-negative T1 progeny of the line B-1 with the *BoSRK3* gene mutation had been segregated out. In addition, five F1 plants of the C-4 × WT were segregated out with the *BoMS1* gene and *BoSRK3* gene mutation but were transgene-free (Supplementary Table 2).

## Discussion

Cabbage is characterized by a typical sporophytic self-incompatibility^20^, while flowering induction also has an obligate requirement for exposure to a prolonged period at low temperatures (vernalization)^27^. Because of the strong self-incompatibility, artificial self-pollination (at the bud stage) has to be performed to reveal the mutant phenotype after treatment with physical or chemical mutagens. This essential process is very laborious and limits the size of the population that the breeder can handle. Genome-editing tools have become well developed in recent years, providing a breakthrough in crop improvement. TALENs have been used to successfully induce site-specific mutations in the *FRIGIDA* gene in cabbage in our laboratory^4^. However, as a species recalcitrant to genetic engineering transformation^28^ until now, the gene editing of cabbage using the CRISPR/Cas9 system has not been published, except for a report on the internet about a meal, which comprised CRISPR/Cas9-edited cabbage (probably a cabbage mustard, judging from the published picture) at Umea University, Sweden. In this study, the *BoPDS* gene, the self-incompatibility gene *BoSRK3*, the *BoMS1* gene associated with male sterility, and some of their paralogous genes were successfully knocked out using an array of sgRNA-tRNA units designed to express more than one sgRNA and showed obvious mutant phenotypes in the T0 generation. In addition, the CRISPR/Cas9 system can target multiple sites or multiple genes in a single transformation event and produce homozygous knockouts, even in the T0 generation, as reported in other species^7–10,13,14^.

The Cas9 protein requires only a PAM (NGG) adjacent to the sgRNA homology region to result in efficient Cas9 binding and DSBs, but there are many possible PAM sites in a gene, and variable gene modification rates (from 0% to 100%) associated with different sites have been frequently reported^12,26,29,30^. Many online tools and resources for computer-based sgRNA design for high activity site prediction have been developed^31^. However, a low relevance has frequently been found between the sgRNA efficiency predicted bioinformatically and that assessed using protoplast or leaf transient expression assays^12,32^. In this study, we manually selected sgRNA target sequences based on the requirements of the Cas9 system and expressed multiple sgRNAs from a single tRNA-sgRNA transcript array to target different sites or genes. Of the T0 plants, 68% were found to contain a *BoPDS* gene mutation based on an abnormal phenotype. On the basis of PCR fragment direct sequencing, 100% of the T0 plants were shown to harbor a *BoSRK3* gene mutation, and 27.78% of the T0 plants were shown to harbor *BoMS1* and *BoSRK3* gene mutations simultaneously when both genes were targeted. However, a few invalid sites (with no mutation detected) were found in the *BoPDS* and *BoSRK3* genes (Figs. 2b and  3a) and are why more than one sgRNA is frequently assembled to simultaneously target multiple sites of a gene to guarantee successful gene modification^9,10,15,26,29,30^.

Plant genomes typically exhibit polyploidy and have redundant genes and extensive gene family networks; therefore, multiple sgRNA designs are required for successful trait modification^13,14^. The most common way to edit multiple sites or multiple genes is to combine multiple sgRNAs, each under the control of its own promoter, into a single construct^6,8,29^. Recently, an endogenous tRNA-processing system to edit multiple genes was accomplished in rice, maize, wheat, cotton, *Arabidopsis*, *Dictyostelium*, and *Drosophila*^9–13,33,34^. In addition, the tRNA^gly^ originating from plants can efficiently process the tRNA-sgRNA transcript in mammalian cells, suggesting that the processing of tRNAs is highly conserved in different organisms^11^. In *Drosophila* and wheat, high site mutation efficiency could be obtained when the guiding sequence was inserted at a location that was proximal to the upstream Pol III promoter in the tandemly arrayed tRNA-sgRNA architecture^11,12^. However, a comparable mutation frequency in rice was also detected even when the guiding sequences were inserted at different locations in the tRNA-sgRNA architecture^9^. In this study, higher site mutation efficiency was obtained when the guiding sequence was inserted at a location that was distal to the upstream Pol III promoter (Figs. 2b, 3a, 4b and 5b), a finding that was similar to that reported in cotton^13^. In addition, similar results were also found for tobacco *PDS* and *Sulfur* (*Sul*) gene modification (unpublished results).

Recently, a report described high-frequency off-target mutagenesis induced by CRISPR/Cas9 in *Arabidopsis*^35^, a result that was in contrast to two earlier reports in *Arabidopsis*^30,36^. This inconsistency could be because too few mutants sample were performed off-target analysis in the previous studies, and different promoters were used to drive Cas9 expression, since an increased dose of the Cas9/sgRNA complexes significantly enhances the frequency of off-target mutations^35^. In this study, we did not analyze the potential off-target loci following the PAM sequences within the cabbage genome, but those sites in the paralogous genes *Bol016089* and *BoSLG3*, which were highly homologous to the target sites in the *BoPDS* and *BoSRK3* genes, respectively, were examined. The sgRNAs can accommodate mismatches varying by as many as five nucleotides in the 5′ upstream region, but the PAM-proximal “seed” region comprising 10–12 bp is crucial to the Cas9 cleavage activity, and mismatches in this region usually lead to a decrease in or even complete abolition of the target cleavage activity^37^. In *Brassica napus*, no mutations were detected in the putative off-target sites as a result of one to three mismatches, which were found in the “seed sequence” of potential off-target sites^15^. In this study, there were one to four mismatches in the “seed sequence” for the A, B, and C putative sites of the *BoSLG3* gene, and, as expected, no mutations were detected in the putative off-target sites (Fig. 4c). However, mutations were detected at the putative C and D sites of the *Bol016089* gene with one mismatch, but it was out of the “seed sequence” of the guide sequence (Fig. 4a, b). The frequency of off-target mutations induced by CRISPR/Cas9 is typically well below that caused by chemical and physical mutagenesis^1^. In addition, unlike CRISPR-based gene therapy in humans, any off-target modification in plants can be removed by segregation, if necessary.

Male sterility can guarantee high-purity F_1_ hybrid cabbage seeds, which have recently become economically significant, and male-sterile parents are used for cabbage hybrid breeding by plant breeders in some countries^19^. However, labor costs for F_1_ hybrid seed production based on the male-sterile approach cannot be reduced or may even be increased due to the self-incompatibility in the maintainer line, the male-sterile line, and the paternal line. *SRK* is one member of the *S*-locus gene family involved in the regulation of cabbage self-incompatibility. The mutation of the *SRK* gene resulted in a self-compatible line in *B. oleracea* and *Brassica rapa*^38,39^. In this study, knockout of the *BoSRK3* gene completely abolished the self-incompatibility of the cabbage self-incompatible line “F416”, the self-compatibility index of the *BoSRK3* gene mutants at the flowering stage were clearly higher than in the wild type “F416”; the mutants had become completely self-compatible lines (Fig. 3). Thus, direct modification of the *SRK* gene in the male-sterile parent, the maintainer line and the paternal line using the CRISPR/Cas9 system, to reduce the costs of cabbage hybrid seed production, is obviously a more efficient way than the backcrossing strategy using rare natural self-compatible mutants.

Currently, ogura cytoplasmic male sterility (CMS) from radish (*Raphanus sativus*) accounts for the vast majority of *B. oleracea* male-sterility types^40,41^. It is well known that the prevalence of a single cytoplasm in commercial crop cultivars has potential risks due to the associated low genetic diversity; the Texas cytoplasm, which was widely used as a source of CMS to produce maize hybrid cultivars, resulted in maize southern corn leaf blight and yellow leaf blight epidemics in 1970–1971 in the USA^42^. Therefore, to reduce the risk of a single cytoplasm in cabbage hybrid breeding programs, the male-sterile cabbage genetic resources can be expanded by mutating the nuclear genes regulating microsporogenesis, stamen development, or microgametogenesis.

In this study, a biallelic mutation at the D site of the *BoMS1* gene in the C-4 plant induced complete male sterility with no pollen grains produced in the anther locules of the sterile mutant (Fig. 6b), and with the phenotype similar to the mutants of the *MS1* gene in *Arabidopsis*, rice, and pepper^22,23,25^. The *ms1* mutant exhibited no defect with respect to the vegetative phenotype and flower organs with the exception of the anther in *Arabidopsis*, rice and pepper^22,23,25^. As expected, the C-4 mutant of the *BoMS1* gene exhibited complete male sterility without any morphological defects. However, male sterility controlled by recessive nuclear genes is difficult to use due to problems encountered in its maintenance, and 100% male sterility was not obtained. In 2002, strategies to obtain full maintainer lines for the male-sterile parents controlled by recessive nuclear genes were proposed by Perez-Prat and van Lookeren Campagne^43^, and commercial applications of the recessive male-sterile lines have been achieved in maize and rice based on these strategies^44,45^. The C-4 male-sterile mutant is highly cross compatible with the maintainer line (wild type with same S-allele) (Fig. 6c). The results indicate that the propagation of the cabbage male-sterile line is economically feasible by the simultaneous mutation of the *BoSRK* gene and the male-sterility gene, indicating that an F_1_ hybrid cabbage seed production system from the *BoMS1* male-sterile line could be constructed in the near future using the same strategy as in maize and rice^44,45^.

## Conclusions

Taken together, our results demonstrated that the tRNA-processing system is capable of targeting multiple sites of a gene or multiple genes in a single transformation event and can produce homozygous or biallelic mutations at multiple loci in the T0 generation that are inherited by the next generation. In addition, we have also produced simultaneous mutations of the allene oxide synthase gene (*BoAOS)* (a gene involved in jasmonic acid biosynthesis) and the *BoSRK3* gene to develop a jasmonic acid-regulated cabbage male-sterile line using the tRNA-processing system (unpublished results). Therefore, we conclude that the multisgRNA-expression-based tRNA-processing system provides a powerful tool to study gene function and to achieve trait stacking in cabbage.

## Materials and methods

### Vector construction

According to the principle of the endogenous tRNA-processing system reported by Xie et al.^9^, a polycistronic tRNA-sgRNA-expressing cassette sequence was synthesized by the Shanghai Xuguan Biotechnology Co., Ltd, Shanghai, China. A "G" nucleotide was inserted between the *Arabidopsis U6-26* promoter and the first glycine-tRNA sequence of *Arabidopsis* to meet the transcription initiation requirement of the *U6* promoter. In addition, the sgRNA structure was modified by extending the duplex length by ~5 bp and mutating the fourth thymine of the continuous sequence of thymines to cytosine, since this modification was reported to significantly improve the knockout efficiency in human cells^46^. Four tRNA-sgRNA units were assembled into the sequence. *Bbs*I, *Bsa*I, *Bsm*BI, and *Bfu*AI sites were inserted between the glycine-tRNA and sgRNA scaffold sequences to facilitate the target sequence cloning (Supplementary Figure 1). Complementary oligos of targeted sites were ligated into the *Bbs*I, *Bsa*I, *Bsm*BI, and *Bfu*AI sites of the tRNA-sgRNA-expressing cassette as described at http://www.genome-engineering.org/crispr/. All of the pairs of partially complementary oligos for the target site with 4 nt overhangs are listed in Supplementary Table 1.

The Cas9 coding sequence in the pYLCRISPR/Cas9P35S-B vector^8^ as a *Nco*I and *Bam*HI fragment was cloned into the *Nco*I and *Bgl*II sites of the pCA13Bar-35S binary vector (with a *Bar* marker gene, developed by our laboratory) to generate the pCACas9 vector. Four target sites were selected for each gene to generate U6-26::tRNA-sgRNA-PDS-ABCD (*BoPDS* gene) and U6-26::tRNA-sgRNA-SRK-ABCD (*BoSRK* gene)-expressing cassettes with a B*am*HI site at the 5′ and an *Eco*RI site at the 3′ ends. However, the U6-26::tRNA-sgRNA-MS1-ABCD (*BoMS1* gene) had an *Xba*I site at the 5′ and a *Bam*HI site at the 3′ ends. The U6-26::tRNA-sgRNA-PDS-ABCD- and U6-26::tRNA-sgRNA-SRK-ABCD-expressing cassettes were ligated into the *Bam*HI and *Eco*RI sites in pCACas9 to obtain the pCACas-tRNA-sgRNA-PDS-ABCD and PCACas-tRNA-sgRNA-SRK-ABCD vectors, respectively. For simultaneous double gene editing of the *BoSRK* and *BoMS1* genes, the sgRNA-expressing cassettes for the modified *BoMS1* gene were ligated into the *Xba*I and *Bam*HI sites of the PCACas-tRNA-sgRNA-SRK-ABCD vector to generate the PCACas-tRNA-sgRNA-SRK-ABCD/MS1-ABCD vector.

### Cabbage transformation

The self-incompatible line “F416” (*SRK3* haplotype) was used in this study (from stocks developed in our laboratory). A modified version of the transformation method described by Bhalla and Singh^47^ was used. Briefly, hypocotyls from 7- to 10-day-old seedlings were precultured on callus initiation media (MS, 0.05 mg/L 1-naphthaleneacetic acid (NAA), 3 mg/L 6-benzylaminopurine (6-BAP), 30 g/L sucrose and 6 g/L agar) for 2 days before inoculation and coculture with *Agrobacterium tumefaciens* EHA105. The preincubated hypocotyls were soaked in *Agrobacterium*-infection buffer (MS and 30 g/L sucrose; pH 5.8–5.9) for 15 min and transferred to the cocultivation media (MS, 0.05 mg/L NAA, 3 mg/L 6-BA, 30 g/L sucrose, and 6 g/L agar) in the dark for 48 h at 25 °C. The explants were subsequently transferred to callus- and shoot-induction media (MS, 0.05 mg/L NAA, 3 mg/L 6-BA, 30 g/L sucrose, 6 g/L agar, + 4 mg/L phosphinothricin (PPT; for *bar* selection), and 400 mg/L carbenicillin (Cb)). When the regenerating shoots reached 1–2 cm in height, they were transferred to rooting media (MS, 0.1 mg/L NAA, sucrose, 6 g/L agar, 4 mg/L PPT, and 400 mg/L Cb) to obtain transgenic cabbage plants.

### Detection of the mutations

Genomic DNA was extracted from individual T0, T1 and F1 plants using cetyltrimethylammonium bromide as previously described^48^. The CRISPR/Cas9 T-DNA-positive transgenic lines were genotyped for mutations using primers flanking the target sequence. The mutation efficiency was counted as described by Ma et al.^49^. The site mutation types were detected through target region cloning and sequencing. All the detection primers are presented in Supplementary Table 1.

### Self-compatibility or cross-compatibility investigation of the *BoSRK* gene mutant plants

The flower buds were covered with a bag one day before anthesis to prevent uncontrolled cross-pollination and pollinated with pollen from the same plant or a wild-type plant with the same S-allele on the day of flowering, with self- or cross-pollination at the bud stage performed as the control. For fluorescence microscopy observation of the pollen germination on the stigma, the pistils were removed from the buds 5 h after the pollen was applied to the stigma and fixed in acetic alcohol (ethanol: acetic acid, 3:1 (v/v)) for 5 h at room temperature. The fixed pistils were hydrolyzed in 2 M NaOH for at least 2 h at 60 °C and then cleared by incubation with 50 mM potassium phosphate solution several times. They were subsequently stained with decolorized aniline blue solution (0.1% aniline blue in 2% K_3_PO_4_) for 2 h. Finally, the stained pistils were mounted on glass slides with 50% glycerol and observed using a UV-fluorescence microscope (Leica CTR5000, Wetzlar, Germany). The self-compatibility index was calculated using the following formula: self-compatibility index = number of seeds/number of flowers. At least three replicates of each treatment were used in the experiment.

### Floral organ and cytological analysis of the *BoMS1* gene mutant plants

On the day of flowering, individual flowers were cut from the inflorescence using a scalpel to observe the structures of the stamen, anther, and pistil under a stereomicroscope. Buds (approximately 3 mm long) from the *BoMS1* gene mutant and the wild type were fixed overnight in 50% ethanol, 5% acetic acid, and 3.7% formaldehyde in water, dehydrated through an ethanol series (30%, 50%, 70%, 80%, 90%, 100%), and embedded into paraffin blocks^50^. Cross-sections were cut, approximately 2–3-μm-thick, stained in hematoxylin, and photographed using a Leica CTR5000 microscope.

## Electronic supplementary material

Supplementary Information
