# Supplementary material for: CRISPR/Cas9-mediated multiple gene editing in Brassica oleracea var. capitata using the endogenous tRNA-processing system
Source: Hortic Res. 2019 Feb 1;6:20. doi: 10.1038/s41438-018-0107-1 (PMC6355899; doi:10.1038/s41438-018-0107-1)
Supplement: Supplementary file 1 — Supplementary Information [file 41438_2018_107_MOESM1_ESM.pdf]

Corresponding author

Correspondence to Hongyuan Song and Xuesong Ren

Supplementary Table 1 Primers used in this study.

Primer name	Sequence（5'-3'）	Purpose
SgSRK-1F	TGCAGGCAATAATCTTGTCCTCCT	*BoSRK3* gene target site assembly
SgSRK-1R	AAACAGGAGGACAAGATTATTGCC	
SgSRK-2F	TGCAGTGGCAGAGCTTCTCGCCAA	
SgSRK-2R	AAACTTGGCGAGAAGCTCTGCCAC	
SgSRK-3F	TGCAGTGGGATCTTTAGAGTGCAT	
SgSRK-3R	AAACATGCACTCTAAAGATCCCAC	
SgSRK-4F	TGCAAACCCTATATCCAACTCCAC	
SgSRK-4R	AAACGTGGAGTTGGATATAGGGTT	
BoSRK3-F	GACATTGTCCGACAGAACCTATG	PCR of the *BoSRK3* genomic sequence
BoSRK3-R	CTTCACTATTATCTGTGAAATTG	
BoSLG3-F	ACGATAATTCTTACACCTCCTGTT	PCR of the *BoSLG3* genomic sequence
BoSLG3-R	TCTTCTAGTATGCCACTATATCGT	
SgPDS-1F	GATTGCAAAGTACCTGGCTGATGC	*BoPDS* gene target site assembly
SgPDS-1R	AAACGCATCAGCCAGGTACTTTGC	
SgPDS-2F	GATTGAAGCAAGAGATGTTCTTGG	
SgPDS-2R	AAACCCAAGAACATCTCTTGCTTC	
SgPDS-3F	GATTGAAACAACGAGATGCTGACA	
SgPDS-3R	AAACTGTCAGCATCTCGTTGTTTC	
SgPDS-4F	GATTACCTGATCGCGTGACTGATG	
SgPDS-4R	AAACCATCAGTCACGCGATCAGGT	
SgMS1-1F	GATTGCCTTTCTAAAACTAGAAGG	*BoMS1* gene target site assembly
SgMS1-1R	AAACCCTTCTAGTTTTAGAAAGGC	
SgMS1-2F	GATTGAGTAGCAAAAGGAGAGCCG	
SgMS1-2R	AAACCGGCTCTCCTTTTGCTACTC	
SgMS1-3F	GATTGAGGTTAAGAGAGGCTTCGA	
SgMS1-3R	AAACTCGAAGCCTCTCTTAACCTC	

SgMS1-4F	GATTAAGTTGGTCCTTTCAGCTCG	
SgMS1-4R	AAACCGAGCTGAAAGGACCAACTT	
BoMS1-F1	TGTCGAATCTGATTCGAACAGATC	PCR of the *BoMS1* genomic sequence (site D, site C and site A regions)
BoMS1-R1	AAGGACATGGCCTTGAAGTTCCAC	
BoMS1-F2	CATGGTTGAGAGCAATTTCAACC	PCR of the *BoMS1* genomic sequence (site B region)
BoMS1-R2	AGTTGGTACTATCAATGGAGATG	
BoPDS-F1	AGACAAGAACAAGGCGAAGGACT	PCR of the *BoPDS* genomic sequence
BoPDS-R1	CCGGTTTCATACCAATCTCCATC	
BoPDS-F2	ATGTTCTACCAGCACCCTTAAAC	
BoPDS-R2	AGGTACTCCCTGGAAATGAACAC	
BoPDS-R3	AGTTATTGGAGTGCCCAAACCTG	
BoPDS-F	CCTTTTATTCATTATGCTACCCAG	
BoPDS-R	GAGAAGTGAGCATGCTTTAGTGGT	
Bol016089-F	TGGTGAAGCTCCTTTGCCTCTCG	PCR of the *Bol016089* genomic sequence
Bol016089-R	TAAGAACGCCATCTTCGACCCAT	
Cas9-F	CAAGTACGTGAACTTCCTCTACC	Detection of the transgenic plants
Cas9-R	GCTGGGAAAGGTCGATACGAGTC	

Supplementary Table 2 Transmission of Cas9/gRNA-induced mutations in the F1 progeny of C-4 × WT ('+': positive; '-': negative)

F1 line	*Cas9*	Site D of the *BoMS1* gene			Site B of the *BoSRK3* gene
		T nucleotide deletion	T nucleotide insertion	WT	A nucleotide insertion
1	+	+	-	-	+
2	+	+	-	-	+
3	-	-	-	+	+
4	+	+	-	-	+
5	+	+	-	-	+
6	-	+	-	-	+
7	+	+	-	-	+
8	+	-	+	-	+
9	-	+	-	-	-
10	-	+	-	-	+
11	-	+	-	-	+
12	+	-	+	-	+
13	-	+	-	-	-
14	-	+	-	-	+
15	+	+	-	-	-
16	-	+	-	-	-

17	-	+	-	-	-
18	-	+	-	-	-
19	+	+	-	-	-
20	-	-	+	-	-

Supplementary Figure 1 Detection of a large fragment deletion in *BoPDS* and *BoSRK3* and their paralogous genes. (**A**) No fragment deletion in the *BoPDS* gene was detected in any *BoPDS* gene mutant plants using the primers BoPDS-F and BoPDS-R (see Supplementary Table 1). **(B)** No fragment deletion in the *Bol016089* gene was detected in any *BoPDS* gene mutant plants using the primers Bol016089-F and Bol016089-R (see Supplementary Table 1). **(C)** No fragment deletion of the *BoSRK3* gene was detected in any *BoSRK3* gene mutant plants using the primers BoSRK3-F and BoSRK3-R (see Supplementary Table 1).

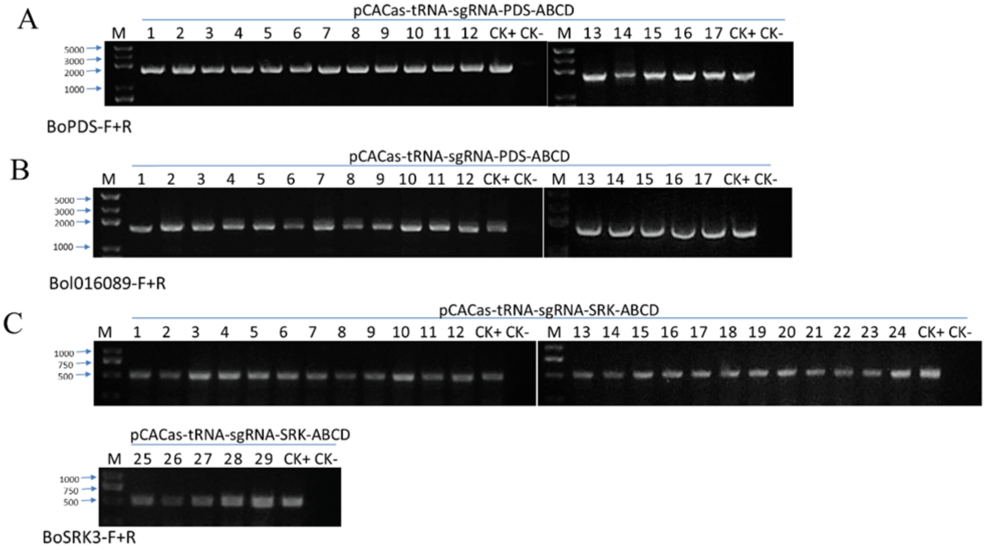

Supplementary Figure 2 Self-pollination of fertile T0 plants with the *BoSRK3* gene mutation without artificial or natural pollination. **(A)** Siliques of the B-2 line. (**B**) Siliques of the B-1 line.

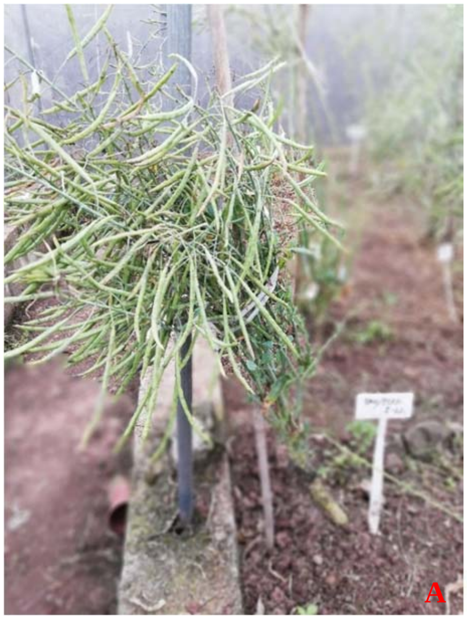

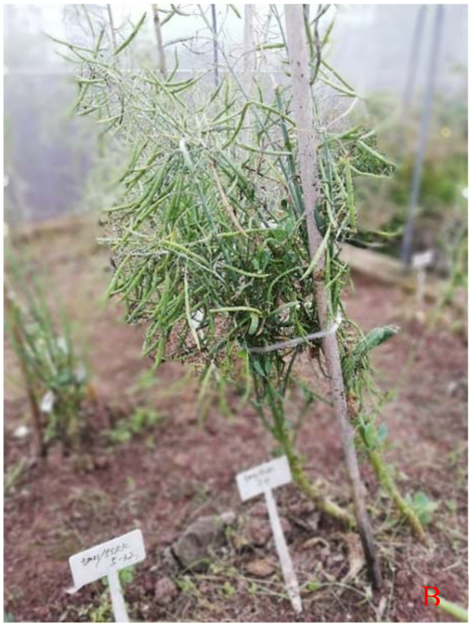

Supplementary Figure 3. Examples of sequencing chromatograms of the T1 and F1 progeny with Cas9-induced mutations. **(A)** A homozygote of the site A mutation (**A1**) and a heterozygote of the site B mutation (**A2**) in the *BoSRK3* gene were detected in the T1 progeny of the B-1 line; New mutations at site B were detected in the T1 generation of the line B-1 (**A3**). **(B)** The ‘T’ nucleotide deletion mutation (**B1**) and the ‘T’ nucleotide insertion mutation (**B2**) detected at the D site of the *BoMS1* gene in the T0 generation (C-4 line) was inherited to the F1 progeny of the C-4 × WT; The ‘A’ nucleotide insertion mutation detected at site B of the *BoSRK3* gene in the T0 generation (C-4 line) was inherited in the F1 progeny of the C-4 × WT (**B3**).

A

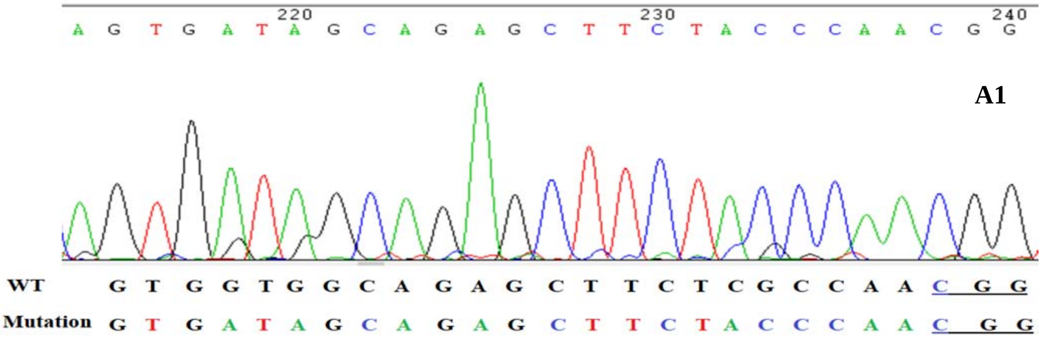

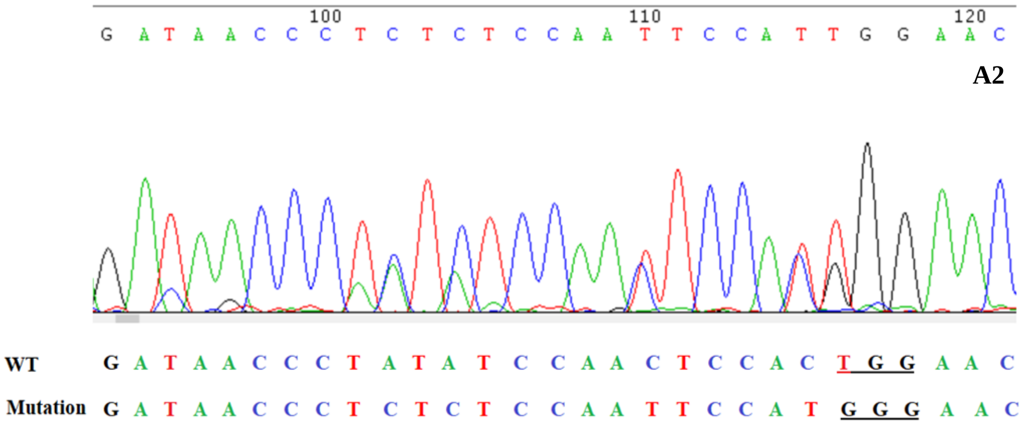
100 110 120
G A T A A C C C T C T C T C C A A T T C C A T T G G A A C
A2
WT G A T A A C C C T A T A T C C A A C T C C A C T G G A A C
Mutation G A T A A C C C T C T C T C C A A T T C C A T G G G A A C

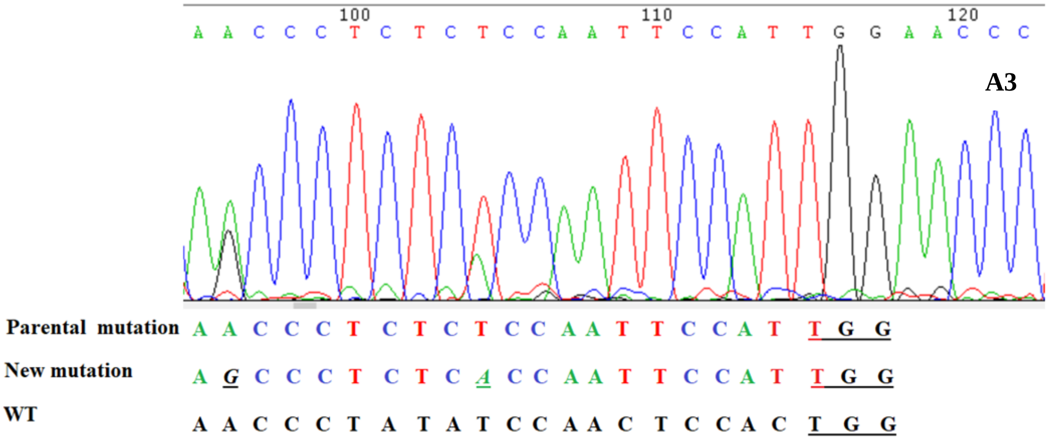
100 110 120
A A C C C T C T C T C C A A T T C C A T T G G A A C C C
A3
Parental mutation A A C C C T C T C T C C A A T T C C A T T G G
New mutation A G C C C T C T C A C C A A T T C C A T T G G
WT A A C C C T A T A T C C A A C T C C A C T G G

B

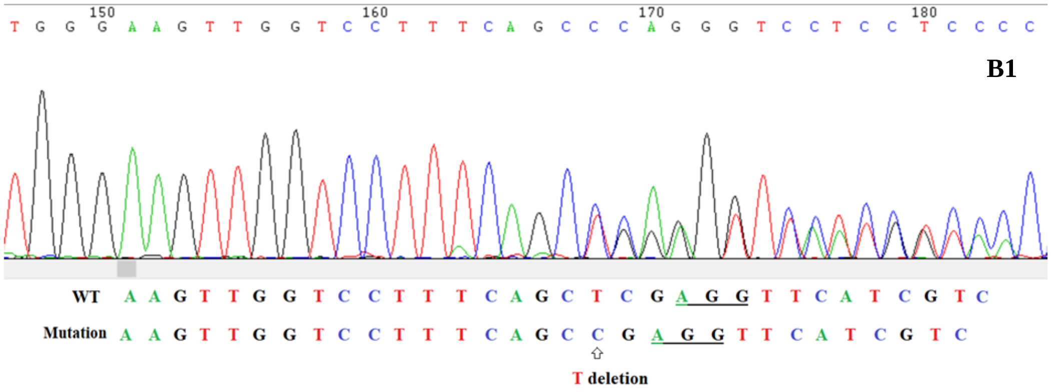
150 160 170 180
T G G G A A G T T G G T C C T T T C A G C C C A G G G T C C T C C T C C C C
B1
WT A A G T T G G T C C T T T C A G C T C G A G G T T C A T C G T C
Mutation A A G T T G G T C C T T T C A G C C G A G G T T C A T C G T C
T deletion

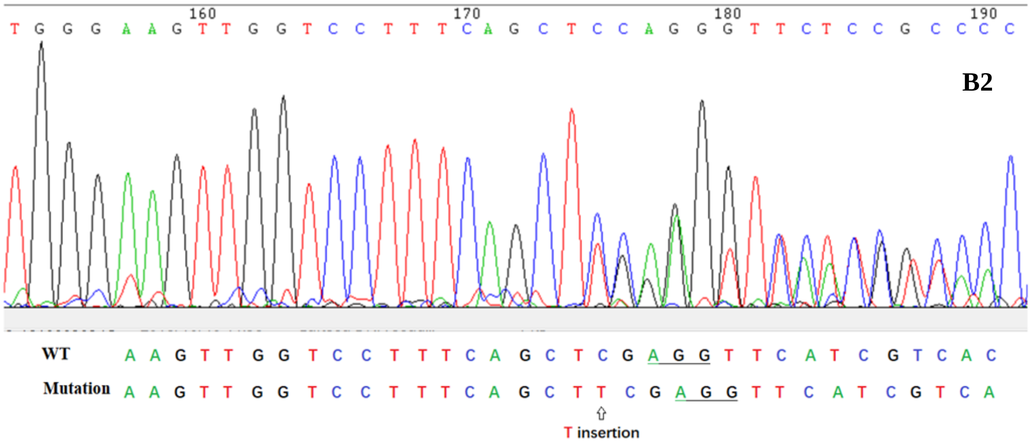

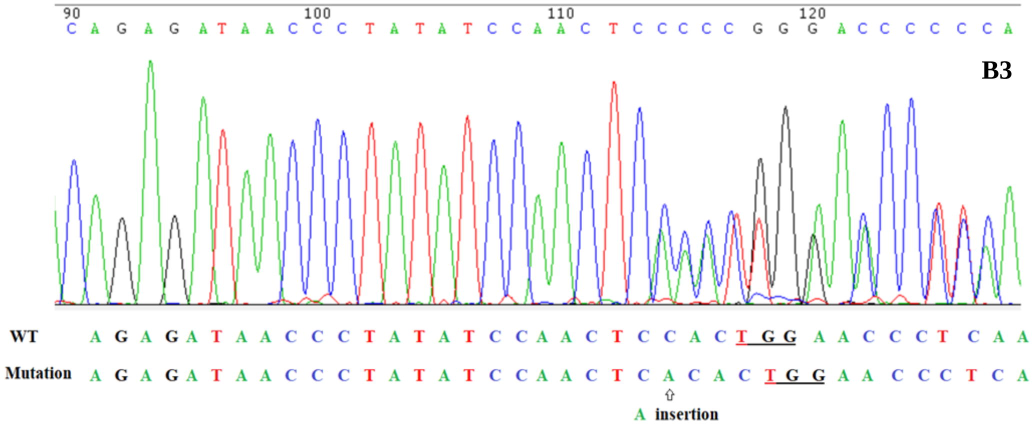

Supplementary data sheet

1. Sequence of tandemly arrayed tRNA-gRNA architecture with the *AtU6-26* promoter. The red sequence represents the *AtU6-26* promoter that drives the tRNA-sgRNA. The green sequence represents *A. thaliana* glycine-tRNA. The blue sequence represents the *Bbs*I, *Bsa*I, *Bsm*BI, and *Bfu*AI restriction sites. The yellow sequence represents the optimized sgRNA scaffold. The orange sequence represents the terminator. The lowercase letters represent the *Bam*HI and *Eco*RI restriction sites.

>AtU6-26::tRNA-sgRNA
ggatccCGTTGAACAACGGAAACTCGACTTGCCTTCCGCACAATACATCATTTCTTCTTA
GCTTTTTTTCTTCTTCTTCGTTCATACAGTTTTTTTTTGTTTATCAGCTTACATTTTCTTG
AACCGTAGCTTTCGTTTTCTTCTTTTTAACTTTCCATTCGGAGTTTTTGTATCTTGTTTC
ATAGTTTGTCCCAGGATTAGAATGATTAGGCATCGAACCTTCAAGAATTTGATTGAAT
AAAACATCTTCATTCTTAAGATATGAAGATAATCTTCAAAAGGCCCCTGGGAATCTGA
AAGAAGAGAAGCAGGCCCATTTATATGGGAAAGAACAATAGTATTTCTTATATAGGC
CCATTTAAGTTGAAAACAATCTTCAAAAGTCCCACATCGCTTAGATAAGAAAACGAA
GCTGAGTTTATATACAGCTAGAGTCGAAGTAGTGATTGAACAAAGCACCAGTGGTCT
AGTGGTAGAATAGTACCCTGCCACGGTACAGACCCGGGTTCGATTCCCGGCTGGTGC
A**GGGTCTTCGTTACAGAAGACCT**GTTTCAGAGCTATGCTGGAAACAGCATAGCAAG

TTGAAATAAGGCTAGTCCGTTATCAACTTGAAAAAGTGGCACCGAGTCGGTGCAACAA
AGCACCAGTGGTCTAGTGGTAGAATAGTACCCTGCCACGGTACAGACCCGGGTTCGA
TTCCCGGCTGGTGCAA**GAGACC**CTGCAGGGTCTCTGTTTCAGAGCTATGCTGGAAAC
AGCATAGCAAGTTGAAATAAGGCTAGTCCGTTATCAACTTGAAAAAGTGGCACCGAG
TCGGTGCAACAAAGCACCAGTGGTCTAGTGGTAGAATAGTACCCTGCCACGGTACAG
ACCCGGGTTCGATTCCCGGCTGGTGCA**AGAGACGGTTACACGTCTCT**GTTTCAGAG
CTATGCTGGAAACAGCATAGCAAGTTGAAATAAGGCTAGTCCGTTATCAACTTGAAA
AAGTGGCACCGAGTCGGTGCAACAAAGCACCAGTGGTCTAGTGGTAGAATAGTACCC
TGCCACGGTACAGACCCGGGTTCGATTCCCGGCTGGTGCA**AATCGCAGGTGTTACA**
ACCTGCATTCGTTTCAGAGCTATGCTGGAAACAGCATAGCAAGTTGAAATAAGGCTA
GTCCGTTATCAACTTGAAAAAGTGGCACCGAGTCGGTGCTTTTTTTTTGTCCCTTCgaatt
c

2. Partial sequence of *BoSRK3* gene covering the target region. The target sites (B, D, A, and C sites in turn) are highlighted in green.

>BoSRK3

TTGTATCTCCCGGTGATGTCTTCGAGCTCGGTTTCTTCAAAACCACCTCAAGTTCTCGT
TGGTATCTCGGTATATGGTACAAGACATTGTCCGACAGAACCTATGTATGGATTGCCAAC
AGAGATAACCCTATATCCAACTCCACTGGAACCCTCAAAATCTCAGGCAATAATCTTGT
CCTCCTTGGTGACTCCAATAAACCTGTTTGGTCGACGAATCTAACTAGAAGAAGTGAG
AGATCTCCAGTGGTGGCAGAGCTTCTCGCCAACGGAAACTTCGTGATGCGAGACTCCA
ACAACAACGATGCAAGTCAATTTTTATGGCAAAGTTTCGATTACCCTACAGATACTTTG
CTTCCTGACATGAAACTGGGTTACGACCTAAAAACAGGGCTGGACAGATTCCTTACAT
CATGGAGAAGTTTAGATGATCCGTCAAGCGGGAATTTCTCGTACAGGCTCGAAACCCG
AAAGTTTCCTGAATTTTATTTAAGGAGTGGGATCTTTAGAGTGCATCGGAGTGGTCCAT
GGAATGGAATCCGATTTAGTGGCATACCAGATGACCAAAAGTTGAGTTACATGGTTTAC
AATTTCACAGATAATAGTGAAGAAGTCGCTTATACATTCCGAATGACCAACAACAGCAT
TTACTCGAGACTGACAGTAAGTTTCTTAGGGCATTTTGAACGTCAGACGTGGAATCCGT
CATTAGGGATGTGGAACGCGTTCTGGTCTTTTATATTGGACTCACAGTGCGATATATACA

3. Partial sequence of the *BoSLG3* gene covered the target region. The putative target sites (B, D, A, C sites in turn) are highlighted in green

>*BoSLG3*

ATGAAAGGCATAAGAAAAACCTACGATAATTCTTACACCTCCTGTTTGCTTGTCTTTTTC
GTCTTAATTCTATTTCGCCCTGCCTTTTCGATCAACACTTTGTCGTCTACAGAATCTCTT
ACAATCTCAAGCAACAGAACACTCGTATCTCCATGTAGTAACTTCGAGCTCGGCTTCTT

CAGAACCAACTCAAGTTCTCGTTGGTATCTCGGGATATGGTACAAGAAATTGTCTAACA
GAACCTATGTATGGGTTGCCAACAGAGATAACCCTTTGTCCAACTCCATTGGAACCCTC
AAAATCTCAGGCAATAATCTTGTCCTCCTTGGTCACTCCAATAGATCAGTTTGGTCGAC
GAATCTTACTAGAGAAAATGAGAGATCCACAGTGGTCGCAGAGCTTCTCGCTAATGGA
AACTTCGTGATGCGAGACGCAAGTGGATTCTTGTGGCAAAGTTTCGATTACCCTACAG
ATACTTTGCTACCAGAGATGAAACTGGGTTACGACCTCAAAACAGGGCTGAACAGGTT
CCTTACATCATGGAGAAGTTCAGATGATCCATCAAGCGGGGATTTCTCGTACAAGCTCG
AAACCCGAAGGCTTCCTGAATTTTATATATCGAGTGGGGTCTTTCTATTGTATCGGAGTG
GACCATGGAATGGAATACGATATAGTGGCATACTAGAAGACCAAAAGTTGAATTACATG
GTGTACAATTTCACAGAGAATAGTGAAGAAGTCGCTTATACATTCCGAATGACCAACA
ACAGCATCTACTCGAGACTGACACTAAGTTTCTCGGGGTATTTTGAACGACAGACGTG
